# The extracellular EXO protein mediates cell expansion in Arabidopsis leaves

**DOI:** 10.1186/1471-2229-9-20

**Published:** 2009-02-13

**Authors:** Florian Schröder, Janina Lisso, Peggy Lange, Carsten Müssig

**Affiliations:** 1 Max Planck Institute of Molecular Plant Physiology, Dept. Willmitzer, Am Mühlenberg 1, 14476 Potsdam – Golm, Germany; 2 Universität Potsdam, Karl-Liebknecht-Str. 24/25, Haus 26, 14476 Potsdam – Golm, Germany; 3 GoFORSYS, Universität Potsdam, c/o Max Planck Institute of Molecular Plant Physiology, Am Mühlenberg 1, 14476 Potsdam – Golm, Germany

## Abstract

**Background:**

The *EXO *(*EXORDIUM*) gene was identified as a potential mediator of brassinosteroid (BR)-promoted growth. It is part of a gene family with eight members in Arabidopsis. *EXO *gene expression is under control of BR, and *EXO *overexpression promotes shoot and root growth. In this study, the consequences of loss of *EXO *function are described.

**Results:**

The *exo *loss of function mutant showed diminished leaf and root growth and reduced biomass production. Light and scanning electron microscopy analyses revealed that impaired leaf growth is due to reduced cell expansion. Epidermis, palisade, and spongy parenchyma cells were smaller in comparison to the wild-type. The *exo *mutant showed reduced brassinolide-induced cotyledon and hypocotyl growth. In contrast, *exo *roots were significantly more sensitive to the inhibitory effect of synthetic brassinolide. Apart from reduced growth, *exo *did not show severe morphological abnormalities. Gene expression analyses of leaf material identified genes that showed robust EXO-dependent expression. Growth-related genes such as *WAK1*, *EXP5*, and *KCS1*, and genes involved in primary and secondary metabolism showed weaker expression in *exo *than in wild-type plants. However, the vast majority of BR-regulated genes were normally expressed in *exo*. HA- and GFP-tagged EXO proteins were targeted to the apoplast.

**Conclusion:**

The *EXO *gene is essential for cell expansion in leaves. Gene expression patterns and growth assays suggest that EXO mediates BR-induced leaf growth. However, EXO does not control BR-levels or BR-sensitivity in the shoot. EXO presumably is involved in a signalling process which coordinates BR-responses with environmental or developmental signals. The hypersensitivity of *exo *roots to BR suggests that EXO plays a diverse role in the control of BR responses in the root.

## Background

Multiple pathways control growth and development. BRs received particular attention when BR-deficient and BR-insensitive mutants were identified [1]. Loss of BR action results in extreme dwarfism. Leaves, internodes, and roots of BR-mutants show reduced size and growth of reproductive organs can be impaired [2]. The growth-promoting effect of BR is largely based on the promotion of cell expansion, though BR may also enhance cell proliferation in leaves [3]. The most prominent direct BR-effect is the modification of gene expression patterns. In fact, BR action requires genomic events, and numerous approaches have identified BR-regulated genes [4,5]. The identified genes and physiological studies suggest that BR controls cell wall modifications, organisation of microtubules and cellulose microfibrils, aquaporin activity, and photosynthesis [2,6]. BR-regulated genes also include putative signalling components, among these the EXO protein (At4g08950) [7,8]. *EXO *gene expression is a strong indicator of BR-responses in vegetative tissues. BR-deficient mutants showed weak *EXO *expression, whereas BR application to the wild-type resulted in elevated *EXO *transcript levels [7]. The BR-hypersensitive *bes1-D *mutant exhibited constitutive *EXO *expression [9]. *EXO *overexpression resulted in stronger shoot and root growth in wild-type plants [7]. However, overexpression of *EXO *in the BR-deficient *dwf1-6 *mutant did not normalize dwarfism [7]. EXO action apparently requires the presence of further BR-dependent factors.

The transgenic line AtEM201 contains a T-DNA insertion in the *EXO *promoter. The *EXO *mRNA level was strongly reduced in these plants. However, the plants did not show an abnormal phenotype [8]. Likewise, inhibition of *EXO *expression by means of RNA interference did not result in an abnormal phenotype [7]. The lack of phenotypic changes in either approach could be due to genetic redundancy, or the *exo *mutant phenotype could become evident only under certain growth conditions. Alternatively, the remaining *EXO *mRNA in the AtEM210 and RNAi lines could be sufficient to maintain proper protein levels [7,8].

Here we report on the characterization of an *exo *knock-out mutant that shows dwarfism. We show that diminished growth of *exo *is due to reduced cell expansion rather than impaired cell proliferation. EXO is an extracellular protein that modifies BR-induced growth responses. Expression profiling experiments identified EXO-regulated genes. The potential molecular mode of action of EXO is discussed.

## Results

### The EXO/EXL protein family

Eight homologous proteins including EXO form a protein family in Arabidopsis (see Additional file 1, Figure S1). Structurally conserved proteins were identified in dicots such as tobacco [10], potato [11], wine grape and black cottonwood, and monocots such as rice and *Sorghum bicolor*, the conifer *Picea sitchensis*, and the moss *Physcomitrella patens*. The genome of the soil bacterium *Solibacter usitatus *also encodes a putative PHI1/EXO-like protein of 317 amino acids (see Additional file 2). No further homologs were identified in bacteria, archaea, fungi, animals, and protists. A phylogenetic tree is shown in Figure S2. The conserved region comprises almost the complete primary structure of about 300 amino acids (Interpro entry IPR006766; PFAM entry PF04674).

### *EXO *and *EXL *expression patterns

**Figure 1:**
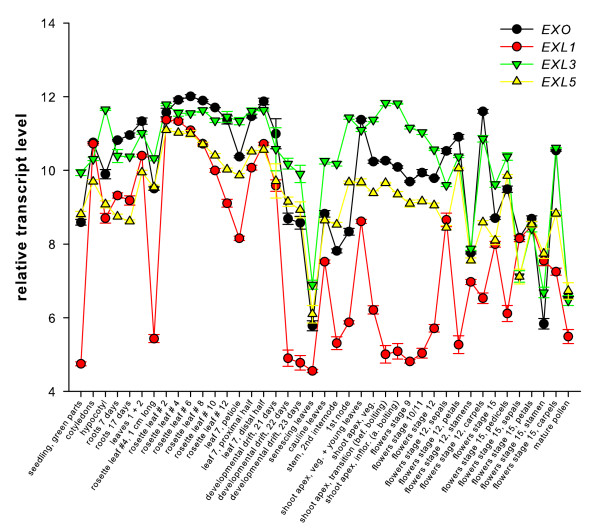
***EXO*, *EXL1*, *EXL3*, and *EXL5 *gene expression in different organs and developmental stages**. Wild-type expression profiles of the development series [12] were downloaded from AtGenExpress and normalized using RMA-Express. The mean and SD of three replicates are shown.

The AtGenExpress development series [12] was used to analyze transcript levels in different organs. Strong *EXO *expression was observed in rosette leaves, cotyledons, and roots. Senescing leaves and pollen displayed little *EXO *expression (Figure 1). Flower organs (i.e., sepals, petals, stamens, and pedicels) had varying mRNA levels. The *EXO-Like1 *(*EXL1*), *EXL3*, and *EXL5 *genes showed expression patterns similar to *EXO *(Figure 1). The Spearman's rank correlation coefficient was calculated for the *EXO*/*EXL *gene pairs (see Additional file 1, Table S1). Expression of the *EXL1 *gene (At1g35140) was closely associated with *EXO *expression. The correlation coefficient accounted for ρ = 0.71 in all profiles and ρ = 0.87 in profiles of vegetative tissues (without flower organs and pollen). *EXL3 *(At5g51550) and *EXL5 *(At2g17230) gene expression correlated positively with *EXO *expression as well (see Additional file 1, Table S1). Associated expression of genes may indicate a common role in physiological processes or pathways. Interestingly, *EXL1*, *EXL3*, and *EXL5 *expression was also induced by BR (see Additional file 1, Table S2). In contrast, mRNA levels of the remaining four *EXL *genes (i.e., *EXL2 *[At5g64260], *EXL4 *[At5g09440], *EXL6 *[At3g02970], and *EXL7 *[At2g35150]) were not altered upon BR-application, and were not associated with *EXO *expression (see Additional file 1, Figure S3, Figure S4, Table S1).

### The EXO protein is required for shoot and root growth

**Figure 2:**
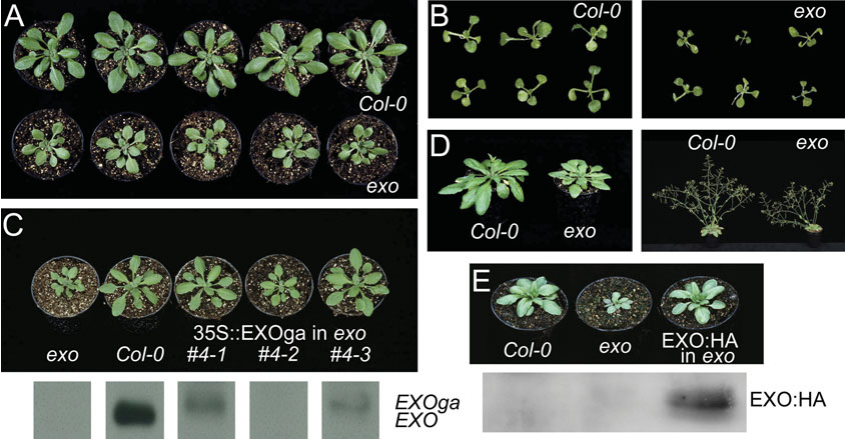
**Phenotype of the *exo *mutant**. A: Wild-type and *exo *(35 days after sowing). B: Wild-type and *exo*. Plants were grown in 0.5 × MS medium for 16 days under aseptic conditions. C: Wild-type, *exo*, and three plants of the segregating transgenic *exo *line # 4 (T2 generation) carrying the 35S::EXOga construct (29 days after sowing). Complemented *exo *plants (# 4-1 and # 4-3) showed detectable *EXOga *transcripts as demonstrated by northern-blot analysis. RNA samples from left to right as indicated above (*exo*, WT, 35S::EXOga plant #4-1, #4-2, and #4-3). Similar results were obtained for independent transgenic lines. D: Wild-type and *exo *(40 and 49 days after sowing). E: Wild-type, *exo*, and homozygous transgenic *exo *plant carrying the 35S::EXO:HA construct. Complemented *exo *plants showed detectable EXO:HA protein as demonstrated by Western analysis. Protein samples from left to right as indicated above (Col-0, *exo*, 35S::EXO:HA in *exo*). A monoclonal anti-HA antibody was used. Similar results were obtained for independent transgenic lines.

An *exo *knock-out mutant (SALK 098602) [13] was identified which carries a single insertion in the *EXO *coding sequence (see Additional file 1, Figure S1). Growth of *exo *plants was reduced in soil and in synthetic medium (Figure 2). Fresh weight and dry weight of *exo *shoots were diminished in comparison to the wild-type. The *exo *plants produced 50 to 60% of the wild-type fresh and dry weight in six independent experiments (Table 1). In further independent growth experiments, biomass production was reduced to a variable extent. In three experiments, *exo *fresh weight was reduced to approximately 40% of the wild-type level, whereas in three other experiments the mutant produced up to 81% of the wild-type fresh weight (see Additional file 1, Table S3). Differences in biomass production of soil-grown plants may depend on the light conditions which could not be fully controlled in the greenhouse.

**Table 1:** Growth parameters of the *exo *mutant.

	**WT**	***exo***	**WT**	***exo***	**WT**	***exo***
	**Soil 28 d**		**Soil 33 d**		**Soil 35 d**	
Fresh weight [mg]	299	179	672	377	538	311
SD	61	64	146	83	129	88
% WT		60		56		58
						
Dry weight [mg]	22.1	13.4	51.6	30.6	44.8	25.5
SD	0.0	0.0	0.1	0.0	0.1	0.0
% WT		61		59		57
						
	**0.5 × MS 10 d**		**0.5 × MS 15 d**		**0.5 × MS 25 d**	
Fresh weight [mg]	8.3	5.1	37.4	18.3	244	125
SD	0.7	0.8	7.8	4.4	32	47
% WT		62		49		51

Fresh and dry weight was determined from soil-grown plants in three independent experiments (28 d, 33 d, or 35 d after sowing). In addition, fresh weight was determined from plants grown on half-concentrated MS medium in plates (10 d and 15 d) or jars (25 d). Student's t-test *P*-values (wild-type vs. *exo*) were below 0.01 in all experiments.

Root length of plants grown on vertically oriented plates was determined. 14, 21, and 31 d old *exo *roots were 30, 24, and 19% shorter in comparison to the wild-type, respectively (Table 2). Loss of *EXO *function did not affect organ formation. For example, the regular number of flower organs was formed, and fertility was not impaired. The *exo *mutant showed a slight tendency to delayed flowering. The opposite trend was observed in 35S::EXO plants (C24 background [7]; data not shown). An *EXO *overexpression construct (termed 35S::EXOga) was transformed into the *exo *mutant. *EXO *expression under control of the 35S promoter restored the wild-type phenotype (Figure 2C). Expression of an EXO:HA fusion protein under control of the 35S promoter also restored the wild-type phenotype in *exo *(Figure 2E). These results demonstrate that the mutant phenotype was caused by the T-DNA insertion in the *EXO *coding sequence.

**Table 2:** Root length of the *exo *mutant.

	**WT**	***exo***
**14 d**		
Root length [cm]	2.43	1.69
SD	0.84	0.33
% WT		70
		
**21 d**		
Root length [cm]	3.37	2.57
SD	1.06	0.98
% WT		76
		
**31 d**		
Root length [cm]	6.37	5.18
SD	1.09	1.11
% WT		81

Plants were grown in vertically oriented plates on 0.5 × MS medium. Root length was determined 14, 21, and 31 days after sowing. Student's t-test *P*-values were below 0.01 in all experiments.

Another T-DNA insertion line (SALK 098601) was supposed to carry a T-DNA insertion in the *EXO *coding sequence. In fact, PCR using the LBb1 primer (for left border of T-DNA insertion, see ) and an antisense primer for the 3'UTR of *EXO *resulted in a 0.7 kb fragment and confirmed an insertion in the *EXO *coding sequence. However, no homozygous mutant plants were identified though all plants were resistant to kanamycin. Therefore, the mutant was back-crossed with the Col-0 wild-type and the F2 generation was screened for homozygous plants. PCR analysis of 96 plants indicated that none carried an insertion in the *EXO *gene in both chromosomes. This observation may suggest that a second insertion close to the *EXO *gene impaired development of homozygous plants.

### Loss of EXO results in reduced cell size

**Figure 3:**
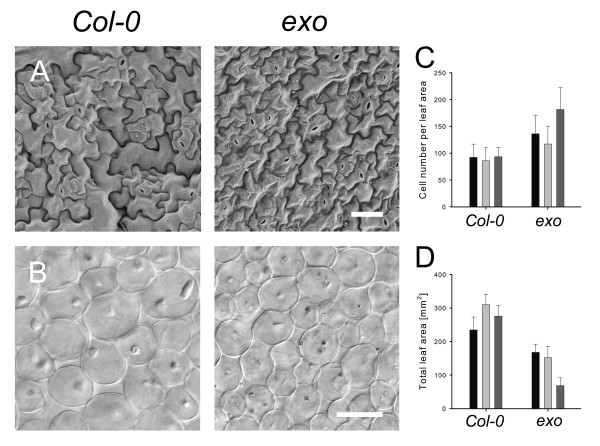
**Cell size and leaf area of *exo *rosette leaves**. Plants grown for 35 days in a greenhouse were subjected to microscopic analyses. A. Scanning electron micrograph (SEM) of abaxial epidermal cells of fully expanded 5^th ^or 6^th ^rosette leaf of wild-type and *exo*. SEM images have the same magnification, the bar represents 40 μm. B. Palisade cells in subepidermal layer of wild-type and *exo*. Bar represents 40 μm. C. Number of palisade cells in subepidermal layer per leaf area (144.000 μm^2^). Data of three independent experiments are shown (mean ± SD). 20 leaves per experiment were analyzed. D. Area of leaf blades. Data of three independent experiments are shown (mean ± SD).

Leaf size is determined by variation in cell size and number. Small leaf size of *exo *could be based on impaired cell expansion, cell proliferation, or a combination of both. Rosette leaves of wild-type and *exo *were subjected to anatomical analysis. Leaf thickness (Table 3) and leaf area were reduced in *exo *(Figure 2, Figure 3D). Epidermal cells of *exo *and wild-type plants were analyzed using scanning electron microscopy (SEM) and found to be smaller in the *exo *mutant (Figure 3A). Histological analysis of subepidermal palisade cells in mature rosette leaves revealed reduced cell expansion in *exo *(Figure 3B). Furthermore, transverse sections of fully expanded leaves showed that palisade and spongy parenchyma cell areas in *exo *were significantly smaller in comparison to the wild-type (Table 3). Thus, EXO is required for cell expansion.

**Table 3:** Leaf thickness, palisade and spongy parenchyma cell areas.

	Leaf thickness [μm]		Palisade parenchyma cell area [μm^2^]		Spongy parenchyma cell area [μm^2^]	
	**WT**	***exo***	**WT**	***exo***	**WT**	***exo***

Mean	683	599	29959	22137	16803	11512
%WT		88		74		69
n	122	365	304	842	404	1138
SD	55	76	5998	8212	5065	6173
t-test		< 0.01		< 0.01		< 0.01
*P*-value						

Plants were grown in soil for 35 days. The 5th or 6th rosette leaves were embedded in 4% agarose and sectioned through the widest part of the blade for transverse sections (n = number of leaves [leaf thickness] or cells [parenchyma cell area]).

Dwarf mutants are frequently characterized by both smaller cells and a decrease in the total number of palisade cells [14,15]. The total number of palisade cells of the 5^th ^or 6^th ^rosette leaf was estimated. The *exo *mutant showed a tendency to fewer cells in comparison to the wild-type, but differences were not consistent in independent experiments (data not shown). Thus, EXO has no major effect on leaf cell number of soil-grown plants.

### EXO is an apoplastic protein

**Figure 4:**
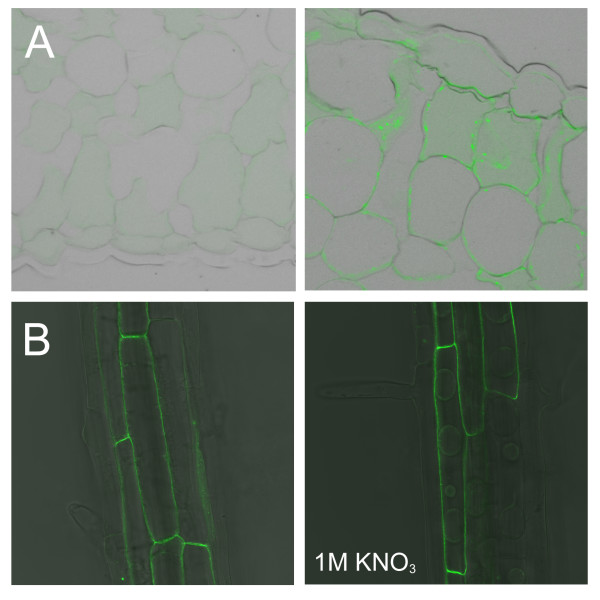
**Localization of EXO:HA and EXO:GFP fusion proteins**. A. Immunological detection of the EXO:HA fusion protein in leaves of stably transformed 35S::EXO:HA plant (right) and wild-type control (left). The bound monoclonal anti-HA antibody was detected using a FITC-coupled secondary antibody. B. Detection of GFP-fluorescence in roots of stably transformed 35S::EXO:GFP Arabidopsis plants in the absence (left) and presence (right) of 1 M KNO_3_.

The subcellular localisation of EXO was analyzed using an HA-tagged EXO protein under control of the 35S promoter. Introduction of the 35S::EXO:HA construct into the *exo *background normalized growth (Figure 2E), demonstrating that the fusion protein is functional. Western-blot analysis showed that the EXO:HA protein was stable in the transgenic plants (Figure 2E). The 35S::EXO:HA construct was transformed into Col-0 wild-type plants, and leaves of transgenic plants were embedded in Technovit 8100. The EXO:HA protein was detected using an anti-HA antibody and a secondary antibody coupled to FITC. The EXO:HA protein was detected in the cell wall (Figure 4A). Only weak background fluorescence was detected in non-transgenic Col-0 plants. Furthermore, a 35S::EXO:GFP construct was stably introduced into Arabidopsis wild-type plants. Green fluorescence was detected in the apoplast. Plasmolysed cells showed fluorescence in the apoplast as well (Figure 4B). These findings are in line with the predictions of sequence analysis tools such as TargetP [16] and Predotar [17] which state that all members of the Arabidopsis EXO/EXL protein family are cell wall-associated. In addition, proteomics approaches also identified the EXO, EXL1, EXL2, EXL3, and EXL4 proteins as part of the cell wall proteome [18-21]. Thus, EXO is an extracellular protein, and the other members of the protein family are also likely to be transported into the apoplast.

### EXO modifies BR responses

**Figure 5:**
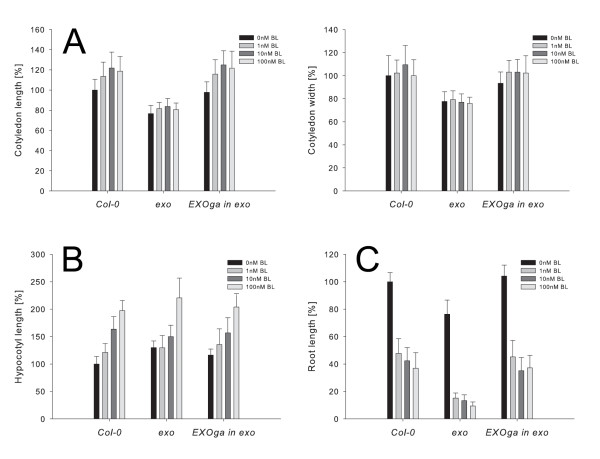
**BR growth responses are modified by EXO**. Wild-type, *exo*, and transgenic *exo *seedlings carrying the 35S::EXOga construct were grown on half-concentrated MS medium for 7 days in the presence of different concentrations of brassinolide (BL). The experiment was carried out independently three times, representative examples of observed responses are shown. 50 plants per treatment and genotype were analyzed. Data are mean ± SD. Cotyledon, hypocotyl, and root growth in response to BL is significantly different in wild-type and *exo *plants (statistics in Additional file 1). A. Cotyledon length and width (mean ± SD; 100% length: 3.3 mm, 100% width 2.8 mm). B. Hypocotyl length (mean ± SD; 100%: 2.7 mm). C. Root length (mean ± SD; 100%: 30.2 mm).

Wild-type, *exo*, and complemented *exo *plants were grown under aseptic conditions in the presence of different brassinolide (BL) concentrations. Cotyledon length, cotyledon width, hypocotyl length, and root length were determined. In the absence of exogenous BL, cotyledon length and cotyledon width were significantly reduced in *exo *compared to wild-type and complemented *exo *plants (Figure 5). BR application resulted in larger cotyledons. However, the relative increase was significantly reduced in *exo *(Figure 5A and Additional file 1). Introduction of the 35S::EXOga construct normalized cotyledon growth. Hypocotyls were slightly longer in *exo *in the absence of BR (t-test, P < 0.001). The *exo *hypocotyls showed a diminished response to BL. Relative hypocotyl elongation was significantly reduced in comparison to the wild-type (Figure 5B and Additional file 1). The observations suggest that EXO is required for BR-induced growth in above ground organs.

The BR-response of roots was also tested. Inhibition of root growth by BL was significantly increased in *exo *compared to the wild-type (Figure 5C and Additional file 1). Introduction of the 35S::EXOga construct normalized *exo *root growth. Thus, loss of EXO results in BR-hypersensitive roots. Since this finding does not hold true for shoot organs, which are less BR-responsive in *exo *plants, the EXO protein may play a diverse and tissue-specific role in the control of BR responses in the root.

### EXO-dependent gene expression

Wild-type and *exo *plants were grown in a greenhouse for 35 days in three independent experiments. Affymetrix ATH1 microarrays were hybridized with labelled cRNA prepared from above ground plant material. Ten genes (including *EXO*) showed significantly weaker expression in the *exo *mutant than in wild-type plants. *WAK1 *(*Wall-Associated Kinase 1*) [22] transcript levels were reduced approximately 2.5-fold in the mutant. Five genes showed significantly stronger expression in *exo *(Table 4). Several other up- and down-regulated genes encode enzymes of the primary or secondary metabolism.

**Table 4:** Genes with altered transcript levels in the *exo *mutant.

**Encoded protein**	**Functional classification**	**log_2 _FC** **(average)**	***P*-value adjusted**
**Weaker expression in *exo***			

At4g08950, EXO		3.5	0.07
At1g21250, WALL-ASSOCIATED KINASE 1 (WAK1)	Signalling, receptor kinase	1.3	0.06
At5g40760, cytosolic glucose-6-phosphate dehydrogenase 6	Glucose metabolism	1.3	0.06
At2g39800, At3g55610, delta1-pyrroline-5-carboxylate synthase	Proline biosynthesis	1.2	0.06
At1g54100, aldehyde dehydrogenase	Oxidation of aldehydes	1.2	0.09
At1g53310, PEP carboxylase 1	Anaplerotic role	1.1	0.06
At4g02480, AAA-type ATPase	Energy-dependent unfolding of macromolecules	1.1	0.07
At5g39320, UDP-glucose 6-dehydrogenase 2	Cell wall precursor synthesis	1.1	0.06
At4g37870, PEP carboxykinase 1		1.0	0.10
At1g17050, solanesyl diphosphate synthase 2	Ubiquinone biosynthetis	1.0	0.08

**Stronger expression in *exo***			

At1g23410, 40S ribosomal protein S27A	Protein degradation, ubiquitin cycle	-1.4	0.07
At4g15680, monothiol glutaredoxin	Thiol metabolism	-1.1	0.09
At5g35480	Unknown	-1.3	0.06
At1g47400	Unknown	-1.2	0.09
At2g25735	Unknown	-1.0	0.06

Wild-type and *exo *plants were grown in soil in three independent experiments and gene expression profiling was carried out by means of Affymetrix ATH1 microarrays. Log_2 _fold changes (log_2 _FC) were determined from Col-0/*exo *signal ratios. A log_2 _FC ratio of one indicates a FC of two. FDR-adjusted *P*-values were calculated using the approach of Benjamini & Hochberg. Filtering criteria: average log_2 _FC ≥ 1 or log_2 _FC ≤ -1 and adjusted *P*-value ≤ 0.10.

The BR-regulated *EXP5 *and *KCS1 *genes were previously reported to be under control of EXO in Arabidopsis C24 plants [7]. Expansins play an essential role in cell wall loosening [23], and the fatty acid elongase, KCS1, catalyses very long chain fatty acid synthesis in vegetative tissues [24]. Real-time RT-PCR analysis demonstrated weaker *EXP5 *and *KCS1 *expression in the *exo *mutant in comparison to Col-0 wild-type plants (Table 5). The differences in *KCS1 *and *EXP5 *transcript levels diminished with increasing plant age (Table 5). Similar development-dependent differences in *KCS1 *and *EXP5 *expression were also observed in plants grown in synthetic medium (data not shown). Weak *KCS1 *and *EXP5 *expression may represent one reason for diminished BR-induced growth in *exo *shoots.

**Table 5:** Real-time RT-PCR analysis of *EXP5 *and *KCS1 *gene expression.

	**WT** 40-dC_T_	***exo*** 40-dC_T_	ddC_T_	**FC**
**10 d**				
***EXP5***	38.4 ± 0.02	37.0 ± 0.02	1.4	2.6
***KCS1***	39.0 ± 0.02	37.9 ± 0.01	1.1	2.1
				
**30 d**				
***EXP5***	37.2 ± 0.19	36.2 ± 0.01	1.1	2.1
***KCS1***	37.7 ± 0.43	36.4 ± 0.01	1.3	2.5
				
**50 d**				
***EXP5***	35.1 ± 0.12	34.4 ± 0.17	0.7	1.6
***KCS1***	32.0 ± 0.12	31.5 ± 0.06	0.6	1.5

Wild-type and *exo *plants were grown in soil. RNA was extracted from rosette leaves of 10-, 30-, and 50 day-old plants. *eIF1α *C_T _values were subtracted from respective C_T _values of the gene of interest resulting in dC_T_. Subsequently, differences were subtracted from an arbitrary value (i.e., 40). Higher numbers indicate higher transcript levels. ddC_T _results from subtracting (40 – dC_T_)_*exo *_from (40 – dC_T_)_WT_. A ddC_T _of one cycle indicates a fold change (FC, wild-type vs. *exo*) of two. Error: SE of gene of interest in three technical replicates. The experiment was carried out independently three times, representative examples of observed values are shown. For sequences of real-time RT-PCR primer pairs see [7].

However, the vast majority of known BR-regulated genes (including genes involved in BR-biosynthesis, BR-catabolism, and BR-signalling) did not show significantly altered transcript levels in *exo*. In line with this finding, we previously showed that *EXO *overexpression does not result in altered transcript levels of BR-regulated genes such as *DWF4 *and *CPD *[7]. Thus, EXO is not a key control element for BR-responsive gene expression in the shoot.

## Discussion

### Structure and subcellular localisation

EXO:GFP and EXO:HA fusion proteins were detected in the apoplast (Figure 4). Other extracellular proteins such as arabinogalactan-proteins (AGPs) are attached to the plasma membrane via a glycosylphosphatidylinositol (GPI) anchor. However, analysis of the EXO primary structure did not reveal a GPI modification site, and plasmolysis experiments showed that the EXO protein was not associated with the plasma membrane (Figure 4). Cell wall proteins are embedded in a polysaccharide matrix and it can be difficult to extract them. Bayer *et al*. [18] identified the EXO, EXL1 (At1g35140), and EXL2 (At5g64260) proteins in extensively washed cell wall preparations. This finding suggests a tight association with the wall. On the other hand, Borderies *et al*. [19] recovered extracellular proteins by washing Arabidopsis cell suspension cultures with salts and chelating agents. They aimed to identify loosely bound cell wall proteins and results were critically evaluated with respect to the integrity of the plasma membrane of the cells. EXO was among the 50 identified proteins. In line with the observation of Borderies *et al*. [19], HA-tagged EXO protein could be extracted readily from 35S::EXO:HA transgenic plants using a standard method for the isolation of soluble proteins (Figure 2E). Thus, a fraction of the EXO protein is loosely bound to the cell wall, though another fraction may strongly interact with cell wall components.

### EXO mediates cell expansion

The *exo *mutant showed reduced leaf size, root length, and biomass production. Reduced leaf size in *exo *is due to diminished expansion of epidermis and parenchyma cells. It was shown that *EXO *gene expression is BR-dependent [7] and under control of the BES1 transcription factor [9]. Different experiments were performed to test the role of EXO in BR-responses. The *exo *mutant showed diminished cotyledon and hypocotyl elongation in response to exogenous BL (Figure 5 and Additional file 1) indicating that EXO is involved in BR-promoted cell expansion. It was shown before that *EXO *overexpression in the BR-deficient *dwf1-6 *mutant did not normalize growth [7]. Similarly, introduction of the 35S::EXOga construct did not normalize the phenotype of the BR-deficient *det2 *mutant ([25], data not shown). Thus, EXO is necessary but not sufficient to mediate BR-promoted growth. Expression profiling experiments demonstrated that EXO controls only a subset of BR-regulated, growth-related genes such as *KCS1 *and *EXP5*. BR-deficiency and BR-insensitivity go along with changes in transcript levels of numerous genes [5]. Since most BR-regulated genes were normally expressed in the *exo *mutant, it appears that EXO does not generally affect BR sensitivity and BR levels in the shoot. Our expression profiling experiments revealed further genes with altered expression levels in *exo *compared with the wild-type (Table 4). For example, At5g39320 and *WAK1 *are expressed at lower levels in the mutant. At5g39320 encodes an UDP-glucose 6-dehydrogenase that could be involved in the synthesis of cell wall precursors. WAK1 is a transmembrane protein containing a cytoplasmic Ser/Thr kinase domain and an extracellular domain which interacts with cell wall pectins [26]. The wall-associated kinases (WAKs) are likely to be involved in signalling between the cell wall and the cytoplasm, and could play a role in development and cell expansion [27].

Thus, EXO is likely to act downstream of the known BR-signalling pathway in the shoot. The protein may mediate BR-induced growth via modifications of cell wall properties and metabolism. BR-hypersensitivity of *exo *roots suggests that EXO plays a root-specific role in the control of BR-responses. The molecular basis of this finding is unknown.

The plant extracellular proteome may comprise 2000 different proteins [21,28]. The PHI1/EXO proteins do not show similarities to proteins with known functions, and thus may have enzymatic or signalling functions that are unknown to date. Our hypothesis is that EXO integrates cellular, metabolic, and/or environmental factors, and feeds this information into an unknown signalling pathway which controls cell wall properties and metabolic pathways. The BR-hypersensitivity of *exo *roots contrasts with the diminished BR-response of *exo *shoot organs. Further studies will address the root-specific phenotype and a potential role of *EXO *in the control of BR-responses in roots.

The *EXO*, *EXL1*, *EXL3*, and *EXL5 *genes showed associated expression in different plant tissues (Figure 1). The similar structure of the EXO, EXL1, EXL3, and EXL5 proteins, the associated expression in different organs, and the common control of expression by BR suggests that all four proteins may play a role in growth control. Genetic redundancy of the *EXO*/*EXL *genes could account for the relative mild phenotypic alterations of *exo *in comparison to BR-deficient or BR-insensitive plants. The generation and analysis of mutants in several *EXO*/*EXL *genes may address this issue.

## Conclusion

The EXO protein represents a class of proteins that occurs widely in the plant kingdom. It is localized to the cell wall and mediates cell expansion. EXO presumably is involved in signalling processes that coordinate BR-responses with environmental or developmental signals.

## Methods

### Screen for mutants

The SALK_098602 line [13] carried a T-DNA insertion in the *EXO *coding sequence and was named *exo*. The DNA insertion site was confirmed by sequencing and is highlighted in Figure S1 (see Additional file 1). Homozygosity of T-DNA insertions was confirmed by PCR on genomic DNA using T-DNA border-specific and gene-specific primers. Impaired gene expression in the knock-out mutant was confirmed using RT-PCR (with primers spanning the respective T-DNA insertion sites) and northern-blot analyses (Figure 2C and data not shown).

### Establishment of transgenic lines

The 35S::EXO overexpression construct, based on a modified pGREEN vector, was described before [7]. A second overexpression construct was established using a Gateway-compatible vector. The *EXO *coding sequence was amplified using the primers EXO_GA_fw 5' CAC CCC TCT TTC ACT ATT ACA CTT TTC CT 3' and EXO_GA_rev 5' GAC CAT AGT AGA GCA AGC CGA C 3'. The PCR fragment was cloned into the pENTR/D-TOPO (Invitrogen, Karlsruhe, Germany) vector, and inserted into the pH7WG2 vector for expression under control of the 35S promoter [29]. The resulting construct was termed 35S::EXOga and used for complementation of the *exo *mutant and transformation of the *det2 *mutant. The pENTR/D-TOPO vector carrying the *EXO *coding sequence was also used to establish the 35S::EXO:GFP and 35S::EXO:HA fusion constructs using the pK7FWG2 [29] and pGWB14 [30] vectors, respectively. All constructs were transformed into Arabidopsis plants using the floral-dip method.

### Growth conditions

Seeds for growth experiments (i.e., wild-type, *exo*, and 35S::EXOga in *exo*) were derived from plants grown in parallel in a greenhouse under identical conditions. Plants were grown in one-half concentrated Murashige and Skoog medium supplemented with 1% sucrose and solidified with 0.7% agar. After two to three days in a cold room (4°C), plants were transferred into a growth chamber with a long day light regime (16 h day, 140 μmol m^-2 ^s^-1^, 22°C; 8 h night, 22°C) and grown in a randomized manner. For monitoring root growth, plants were grown on vertical plates. Alternatively, plants were established in soil (type 'GS-90 Einheitserde', Gebrüder Patzer, Germany). Seeds were allowed to germinate and to grow for two weeks in controlled growth chambers (7 days: 16 h light [140 μmol m^-2 ^s^-1^], 20°C, 75% relative humidity; 8 h night, 6°C, 75% relative humidity; thereafter 7 days: 8 h light [140 μmol m^-2 ^s^-1^], 20°C, 60% relative humidity; 16 h night, 16°C, 75% relative humidity). Subsequently, plants were transferred to long-day conditions in a greenhouse with artificial light (16 h light [high pressure sodium and metal halide lamps], 21°C, 50% relative humidity; 8 h night, 19°C, 50% relative humidity). All genotypes were grown in the same chamber at the same time in a randomized manner.

### Gene expression analysis and protein extraction

RNA for Northern-blot analysis was isolated using the Trizol reagent (Invitrogen). Northern-blot and real-time RT-PCR analysis was performed as described [7]. Generation of labelled cRNA and hybridisation of ATH1 oligonucleotide microarrays were performed using standard protocols in cooperation with Atlas biolabs (Berlin, Germany) as described [7]. Profiles were normalized with RMA (Table 4), MAS5.0/GCOS (Table S2, see Additional file 1), or RMA-Express (Figure 1, Additional file 1: Figure S3, Figure S4, Table S1). Differences between wild-type and *exo *were tested with the LIMMA software package [31] using a moderated paired t-test. FDR-adjusted *P*-values were calculated using the approach of Benjamini & Hochberg.

Protein for Western-blot analysis was isolated using ice cold extraction buffer (50 mM Tris (pH 7.2), 100 mM NaCl, 10% glycerol, Complete Protease Inhibitor Cocktail (Roche, Grenzach, Germany)).

### Leaf cross sections and microscopy

The fifth or sixth leaf of 35-day old plants was used for microscopic analysis. Leaves were embedded in 4% agarose and sectioned at 40 μm through the widest part of the blade for transverse sections using a vibratome (Leica VT 1000S, Bensheim, Germany). Leaf thickness and leaf cell area were analyzed using the cellP software (Olympus, Hamburg, Germany). At least eight leaves per genotype and ten cross sections of each leaf were measured. Cells surrounding the leaf vein were excluded. Images of sections were generated using an Olympus BX41 microscope. Subepidermal cell layers were analyzed using an Olympus AX70 microscope after bleaching of leaves with 1 M KOH for 24 hours. For scanning electron microscopy (SEM), leaf samples were fixed in 3% paraformaldehyde and 0.25% glutaraldehyde in phosphate buffer (pH 7.1) and dehydrated. A gold/palladium (80:20) coat of 2 nm was applied in a cool sputter coater SCD 050 (Bal-tec, Balzers, Liechtenstein). Images of the leaf surface were observed on a LEO 1550 (LEO, Oberkochen, Germany) microscope. GFP-fluorescence was visualized in roots of 27-day old transgenic plants grown in sterile media with a Leica TCS SP5 confocal microscope.

For immunocytochemistry leaves were fixed in 3% paraformaldehyde and 0.25% glutaraldehyde in phosphate buffer (pH 7.1). The samples were dehydrated and infiltrated in Technovit 8100 resin (Heraeus Kulzer, Wehrheim, Germany) according to the manufacturer's protocol. The leaves were sectioned at 1.2 μm using a Leica RM2255 Rotary Microtome and mounted on charged glass slides. The sections were treated with 0.1 M NH_4_Cl in phosphate buffered saline (PBS) for 5 min followed by a washing step in PBS for 5 min. Slides were incubated with 5% bovine serum albumin (BSA) in PBS for 30 min and incubated over night at 4°C in primary antibody (anti-HA, mouse IgG clone 12CA5, Roche) diluted in 5% BSA/PBS at a ratio of 1:60. Three washing steps with 0.1% BSA in PBS for 10 min were followed by one with 1% BSA in PBS for 10 min. Subsequently, the slides were incubated for 1 hour at RT with the secondary antibody (FITC goat anti-mouse IgG (H+L), ZYMED, San Francisco, USA) diluted in 5% BSA/PBS at a ratio of 1:100. After four washing steps with PBS for 10 min, images were generated using an Olympus BX41 microscope.

## Authors' contributions

FS and JL isolated the *exo *mutant, performed the expression analyses, growth assays, and microscopic studies. FS and PL established the transgenic lines. CM designed the study and wrote the manuscript. All authors read and approved the final manuscript.

## Supplementary Material

Additional file 1 **Additional file 1. EXO EXL supp mat.** Figure S1 shows an alignment of primary structures of EXO/EXL proteins. Figure S2 shows a phylogenetic tree of PHI1/EXO sequences. Figure S3 and Figure S4 show EXL2, EXL4, EXL6, and EXL7 expression patterns in the AtGenExpress development series. Table S1 gives correlation coefficients for EXO/EXL transcript levels in the AtGenExpress development series. Table S2 shows the BR-dependent expression of EXO, EXL1, EXL3, and EXL5. Table S3 shows additional biomass data. The supplement to Figure 5 shows the statistical analysis (ANOVA). Click here for file

Additional file 2 **PHI1 EXO sequences – FASTA.** The file provides protein sequences of PHI1/EXO homologs. Click here for file
