# Supplementary material for: The extracellular EXO protein mediates cell expansion in Arabidopsis leaves
Source: BMC Plant Biol. 2009 Feb 13;9:20. doi: 10.1186/1471-2229-9-20 (PMC2661892; doi:10.1186/1471-2229-9-20)
Supplement: Additional file 1 — EXO EXL supp mat. Figure S1 shows an alignment of primary structures of EXO/EXL proteins. Figure S2 shows a phylogenetic tree of PHI1/EXO sequences. Figure S3 and Figure S4 show EXL2, EXL4, EXL6, and EXL7 expression patterns in the AtGenExpress development series. Table S1 gives correlation coefficients for EXO/EXL transcript levels in the AtGenExpress development series. Table S2 shows the BR-dependent expression of EXO, EXL1, EXL3, and EXL5. Table S3 shows additional biomass data. The supplement to Figure 5 shows the statistical analysis (ANOVA). [file 1471-2229-9-20-S1.pdf]

Supplementary material

```
EXO          1 ----MYSLVFKLFLFLSLLQISVSARN-----------------------------------------LASQEPNQFQLLKYHKGALLSG
EXL1         1 ----MASFVMGYFLLFAVAFMCLDAR--------------------------------------------TDKTQDYTSFQYHKGALLTG
EXL2         1 ------MASNYRFAIFLTLFFATAGFS-----------------------------------------AAALVEEQPLVMKYHNGVLLKG
EXL4         1 ------MAYNYRFAILLVLLSATVGFT-----------------------------------------AAALKPP---------VRTLNG
EXL5         1 MSSPATTITFFFFFTLSSFFYITSSLQNNNNNKHTATVNSLNIPSAAAEITLVNPKLPPRSLSLTSSKKFEGSSNLVHLRYHMGPVLSSS
EXL7         1 -------------MSLIIVFFFFS----------------LL--LTSN----------GQ--FFDESKNYEGSSDLVDLQYHLGPVISSP
EXL3         1 ------MHSLPVNLVLTVLTVFLTS------------------PAQVIGYRPYPPKTNGSDQIFDASKKFEGSSNLVRLRYHMGPVLTNN
EXL6         1 --MAMASASSSSSSISVIIFLLLAP-----------------------------------------------LCLS----REPPSQIPNG
consensus    1     . ... . ......... ...                                         .. ........ . . .. .....

                                           exo
EXO         46 KISVNLIWYGKFKPSQRAIISDFITSLTHTSPTSKTLHQPSVATWWKTTEKYYK----------LATPSKNSSPLSLTLGKQIIDESCSL
EXL1        43 DVSINLIWYGKFKPSQRAIVTDFVASLSSSR-RSTMAQNPSVATWWKTVEKYYQ----------FRKMTTTRG-LSLSLGEQILDQGYSM
EXL2        44 NITVNLVWYGKFTPIQRSVIVDFIHSLNSKD-VASSAAVPSVASWWKTTEKYK------------------GGSSTLVVGKQLLLENYPL
EXL4        35 NITLNLIWYGKFTPIQRSIIVDFIRSISS----VTAAKGPSVASWWKTTEKYK------------------TGVSTLVVGKQLLLENYPL
EXL5        91 PINIYVIWYGQWSRPHKSLIRDFLNSISD-----AKAPSPSVSEWWRTASLYTD----------QTG---SNVSRSVLIAGEYSDSKYSH
EXL7        48 VTSLYIIWYGRWNPTHQSIIRDFLYSVSAP----APAQYPSVSNWWKTVRLYRD----------QTG---SNITDTLVLSGEFHDSTYSH
EXL3        67 -ITVHPIWYGTWQKSQKKIIREFINSISA-----VGSKHPSVSGWWKTVQLYTD----------QTG---SNITGTVRLGEEKNDRFYSH
EXL6        38 TLDLSLLWYGQFTPTQKERVHDFIESLNFD---AKEGLDPKVSAWWKVVESYQERFEVKDIYRQKKSNRTVAPRIKVKVVRSYVDEKMKY
consensus   91  ......***............*. *...     . .. *.* .**.....*..          ...   ................ ...

EXO        126 GKSLTDKKIQTLAS--------KGDQR--NAINVVLTSADVTVTGFGMSRCGTHGHARGLGKR-GSKFAYIWVGNSETQCPGQCAWPFHA
EXL1       121 GKSLTEKNLKDLAA--------KGGQS--YAVNVVLTSADVTVQGFCMNRCGSHGTGSGSGKK-GSRFAYIWVGNSETQCPGQCAWPFHA
EXL2       115 GKSLKNPYLRALST--------KLNGG-LRSITVVLTAKDVTVERFCMSRCGTHGSSGSNPRRAANGAAYVWVGNSETQCPGYCAWPFHQ
EXL4       103 GKSLKSPYLRALSS--------KLNAGGARSITVVLTAKDVTVEGLCMNRCGTHGSKSSS----VNSGAYVWVGNSETQCPGYCAWPFHQ
EXL5       163 GQHLTRLTIQEVIAS--AARSASFPVDHKNGMYLVLTSHDVTMQDFCRAVCGFHYFTFPS--MVGYTMPYAWVGQSGKQCPEVCAYPFAL
EXL7       121 GSHLTRFSVQSVIR---TALTSKLPLNAVNGLYLVLTSDDVEMQEFCRAICGFHYFTFPS--VVGATVPYAWVGNSERQCPEMCAYPFAQ
EXL3       138 GKSLTRLSIQSVIKSAVTSRSRPLPVNPKSGLYLLLTADDVYVQDFCGQVCGFHYFTFPS--IVGFTLPYAWVGNSAKLCPGVCAYPFAV
EXL6       125 GKELTMGNGEKLVET--------AIGNMSKVVPVVLLSAQVRAHGVGFCDGTCQHNALAKIKGQKEPRRYIMVSNPEVECPGECAWPFHT
consensus  181 *..*.. ........     . ... .  ......*....*................... . .. ...*..*......**..**.**..

EXO        205 PVYG-----PQSPPLVAPNNDVGLDGMVINLASLLAGTATNPFGNGYYQGP-QNAP---------------LEAASACPGVYGKGAYPGY
EXL1       200 PVYG-----PQSPPLVAPNNDVGLDGMVINLASLMAATATNPFGDGYYQGP-KTAP---------------LEAGSACTGVYGKGSYPGY
EXL2       196 PIYG-----PQTPPLVAPNGDVGVDGMIINLATLLANTVTNPFNNGYYQGP-PTAP---------------LEAVSACPGIFGSGSYPGY
EXL4       181 PIYG-----PQSPPLVAPNGDVGVDGMIINIATLLVNTVTNPSP----------------------------EAVSACTGIFGSGAYPGY
EXL5       249 PG---YMGHGGPGELRPPNGETGVDGMVSVIGHELAEVVSNPLINAWYAGEDPTAP---------------TEIGDLCEGLYGSGGGGGY
EXL7       206 PKPFPGSGFVAREKMKPPNGEVGIDGMISVIAHELAEVSSNPMLNGWYGGEDATAP---------------TEIADLCLGVYGSGGGGGY
EXL3       226 PA-----FIPGLKPVKSPNGDVGVDGMISVIAHEIAELATNPLVNAWYAGPDPVAP---------------VEIADLCEGIYGTGGGGSY
EXL6       207 ADKG-----PRGMTYQPASGEVGADALVIQLATGLADLATNPDLTKSLFKSETTPYNDDVKKNHESSSMYIVDPATKCTRVFGSGAFPGF
consensus  271 ....     .............*.*...............**..............               ......*....*.*.....

EXO        274 AGDLLVDTTTGGSFNAYGANGRKFLLPALYDPTTSACSTMV---
EXL1       269 AGELLVDATTGGSYNVKGLNGRKYLLPALFDPKTDSCSTLF---
EXL2       265 AGRVLVDKTTGSSYNARGLAGRKYLLPAMWDPQSSTCKTLV---
EXL4       238 AGRVLVDKTSGASYNALGLAGRKYLLPALWDPQTSTCKTMV---
EXL5       321 IGQVMRDRE-GKTFNMNGKGGRKFLVQWIWNPNLKACSGPNSVD
EXL7       281 MGSVYKDRW-RNVYNVKGVKGRKYLIQWVWDLNRNRCFGPNAMN
EXL3       296 TGQMLNDHS-GATYNVNGIR-RRYLIQWLWSHVVSYCTGPNALD
EXL6       292 TGRIRVDPITGGAFNSHGINHLKFLIPSIWDPKTKSCWTPM---
consensus  361 .*....*.......*..*......*...........*.... .
```

Figure S1. Primary structures of the EXO and EXL proteins.

Coding sequences were derived from full-length cDNAs. The *EXL6* gene has an intron (data not shown), all other genes feature one continuous exon. The coloured amino acid indicates the position of the T-DNA insertion in the *EXO* gene. Sequences were aligned using the BCM Search Launcher (http://searchlauncher.bcm.tmc.edu/) and BOXSHADE tools (http://www.ch.embnet.org/software/BOX_form.html).

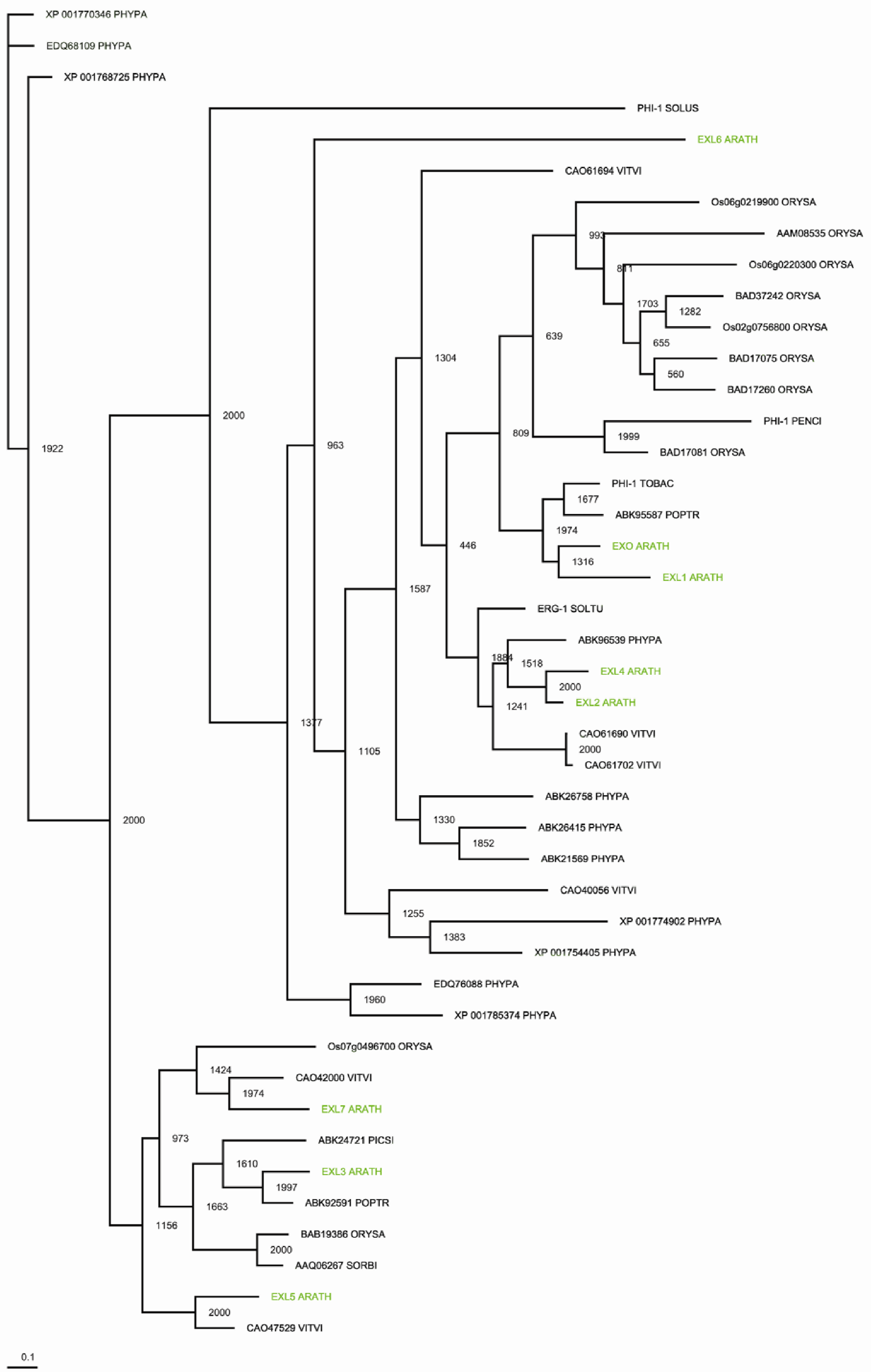
XP 001770346 PHYPA
EDQ68109 PHYPA
XP 001768725 PHYPA
PHI-1 SOLUS
EXL6 ARATH
CAO61694 VITVI
Os06g0219900 ORYSA
AAM08535 ORYSA
Os06g0220300 ORYSA
BAD37242 ORYSA
Os02g0756800 ORYSA
BAD17075 ORYSA
BAD17260 ORYSA
PHI-1 PENCI
BAD17081 ORYSA
PHI-1 TOBAC
ABK95587 POPTR
EXO ARATH
EXL1 ARATH
ERG-1 SOLTU
ABK96539 PHYPA
EXL4 ARATH
EXL2 ARATH
CAO61690 VITVI
CAO61702 VITVI
ABK26758 PHYPA
ABK26415 PHYPA
ABK21569 PHYPA
CAO40056 VITVI
XP 001774902 PHYPA
XP 001754405 PHYPA
EDQ76088 PHYPA
XP 001785374 PHYPA
Os07g0496700 ORYSA
CAO42000 VITVI
EXL7 ARATH
ABK24721 PICSI
EXL3 ARATH
ABK92591 POPTR
BAB19386 ORYSA
AAQ06267 SORBI
EXL5 ARATH
CAO47529 VITVI
1922
2000
2000
963
1377
1304
1587
1105
446
809
639
993
811
1703
1282
655
560
1999
1677
1974
1316
1884
1518
2000
1241
2000
1330
1852
1255
1383
1960
1424
1974
973
1610
1997
1663
1156
2000
2000
0.1

Figure S2. Phylogenetic tree of PHI1/EXO sequence relationships.

EXO homologs were identified using BLAST searches (see Additional file 2). Fragmentary sequences were discarded. Multiple alignments were created by means of the ClustalW2 tool (http://www.ebi.ac.uk/Tools/clustalw2/index.html). Sequences were transformed into the PHYLIB format by means of the GeneDoc software (http://www.nrbsc.org/gfx/genedoc/index.html). The PhyML software package (*Systematic Biology* 2003, 52: 696-704) with the JTT model for proteins was used to create the phylogenetic tree. Branch support was estimated through 2000 bootstrap samples. TreeView (*Computer Applications in the Biosciences* 1996, **12**: 357-358) was used to visualize the tree file.

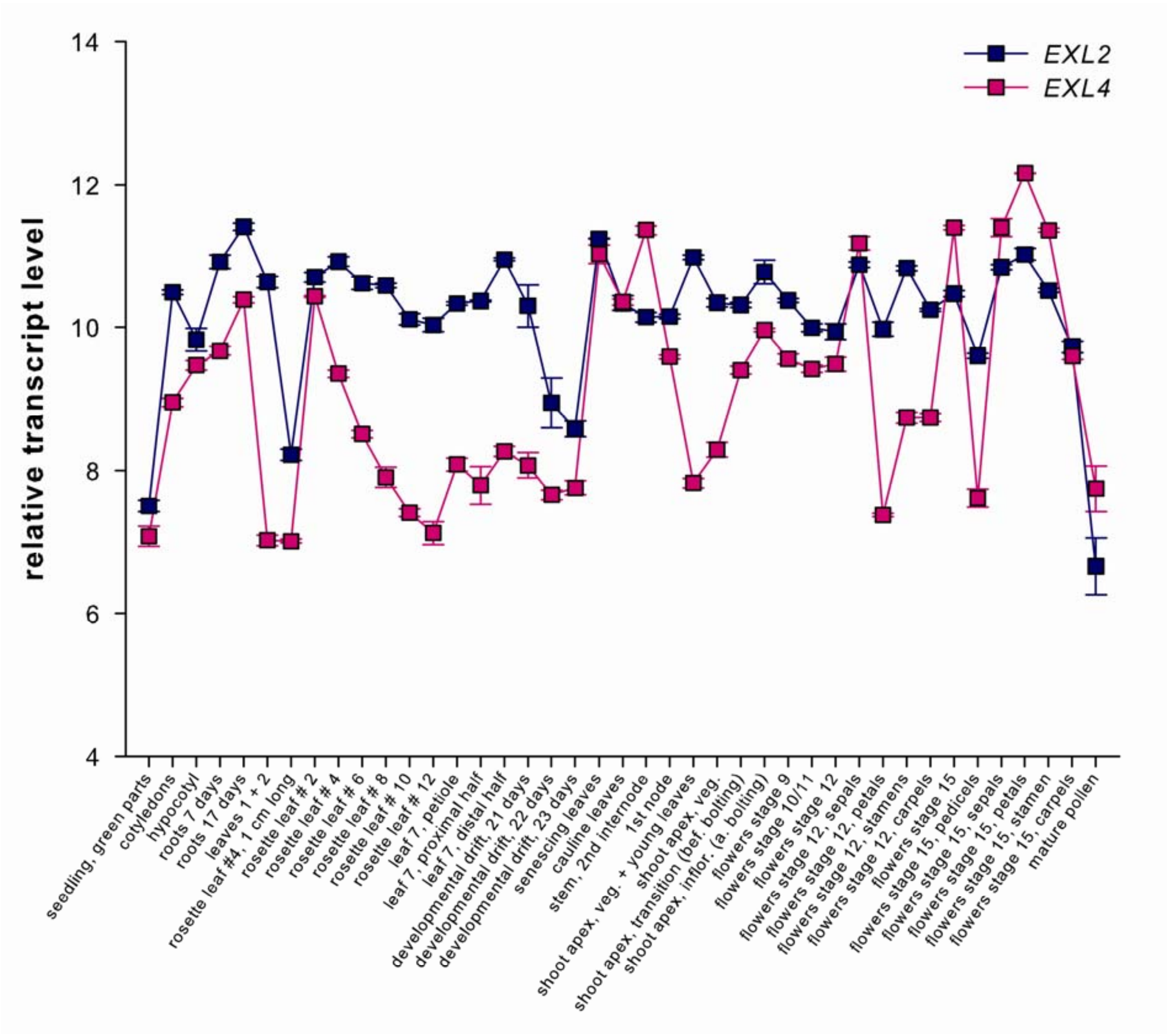

Figure S3. *EXL2* and *EXL4* expression in different organs and developmental stages. Wild-type expression profiles of the development series (*Nat Genet* 2005, **37**: 501-506) were downloaded from AtGenExpress and normalized using RMA-Express (*Bioinformatics* 2003, **19**: 185-193). The mean and SD of three replicates are shown.

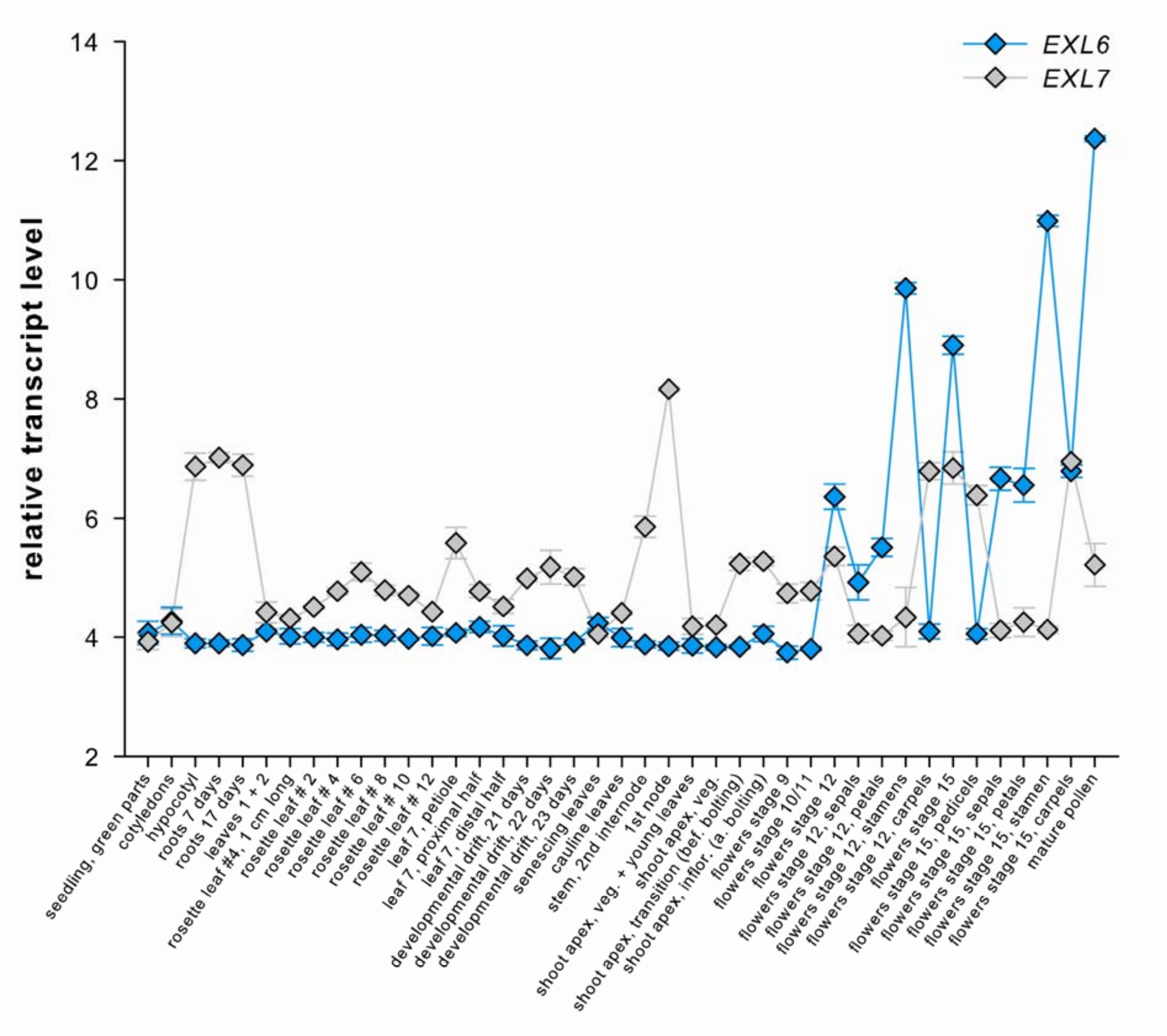

Figure S4. *EXL6* and *EXL7* expression in different organs and developmental stages. Wild-type expression profiles of the development series (*Nat Genet* 2005, **37**: 501-506) were downloaded from AtGenExpress and normalized using RMA-Express (*Bioinformatics* 2003, **19**: 185-193). The mean and SD of three replicates are shown.

Table S1. *EXO* and *EXL* gene expression in different organs and developmental stages.

**All profiles**								
	EXO	EXL1	EXL2	EXL3	EXL4	EXL5	EXL6	EXL7
EXO	-							
EXL1	**0,7070**	**-**						
EXL2	0,2601	0,4843	**-**					
EXL3	**0,7014**	0,3615	0,0547	**-**				
EXL4	-0,2984	0,0174	**0,5080**	-0,2117	-			
EXL5	**0,7592**	**0,5016**	-0,0237	**0,7780**	-0,5258	-		
EXL6	-0,2063	0,0979	0,0841	-0,4850	0,1326	-0,3376	-	
EXL7	0,0789	0,0064	-0,2395	0,2986	0,1322	0,0150	-0,2366	-
**Without flower and pollen**								
	EXO	EXL1	EXL2	EXL3	EXL4	EXL5	EXL6	EXL7
EXO	-							
EXL1	**0,8724**	**-**						
EXL2	0,4487	0,4414	**-**					
EXL3	**0,5818**	0,4676	0,2387	**-**				
EXL4	-0,2094	-0,0031	0,4792	0,1044	-			
EXL5	**0,8388**	**0,7717**	0,1685	**0,6386**	-0,3333	-		
EXL6	0,1868	0,2503	0,1960	-0,0244	-0,1447	0,2534	-	
EXL7	-0,0751	-0,0153	-0,0470	0,2558	0,3718	-0,1239	-0,3748	-

Wild-type expression profiles of the development series (*Nat Genet* 2005, **37**: 501-506) were downloaded from AtGenExpress and normalized using RMA-Express (*Bioinformatics* 2003, **19**: 185-193). Spearman's rank correlation coefficients were calculated for gene pairs based on the transcript levels in all profiles (top) or based on the transcript levels in profiles representing vegetative organs (bottom). Values above 0.5 are in bold type.

Table S2. BR-induced *EXO, EXL1, EXL3*, and *EXL5* gene expression.

	**WT ± EBL 7h**	**WT ± CS 3h**	
	Experiment # 1	Experiment # 1	Experiment # 2
	SLR	SLR	SLR
EXO	1.8	3.2	3.1
EXL1	1.2	4.5	3.3
EXL3	0.8	1.9	1.2
EXL5	0.8	1.1	1.1

	***det2* ± BL 1h**		***det2* ± BL 3h**	
	Experiment # 1	Experiment # 2	Experiment # 1	Experiment # 2
	SLR	SLR	SLR	SLR
EXO	2.0	2.0	2.7	2.6
EXL1	2.7	3.2	4.1	5.5
EXL3			1.1	1.3
EXL5	1.0	1.0	1.0	1.0

Affymetrix expression profiles from AtGenExpress (established by Hideki Goda, RIKEN, Japan) and two further profiles (*FEBS Let* 2004, **563**: 82-86) were analyzed using the stringent settings of the statistical algorithms of the GCOS software as described previously (*Nucleic Acids Res* 2005, **33**: 2685-2696). Wild-type plants (top) and the BR-deficient *det2* mutant plants (bottom) were analyzed. Signal log ratios (SLR) of treatment versus control are shown, a SLR of one corresponds to a two-fold change. *EXO*, *EXL1*, *EXL3,* and *EXL5* transcripts were called 'Present' at least in profiles of BR-treated plants according to the MAS5.0/GCOS algorithm. Change *P*-values (according to GCOS algorithm) were below 0.001 in all experiments. BL: brassinolide; EBL: 24-epibrassinolide; CS: castasterone. SLRs below 0.8 are not shown.

Table S3. Fresh weight of wild-type and *exo* plants.

	**WT**	***exo***	**WT**	***exo***	**WT**	***exo***
	**Soil 32 d**		**Soil 35 d**		**Soil 35 d**	
Fresh weight [mg]	517	202	1268	520	701	263
SD	47	33	303	126	31	50
**% WT**		**39**		**41**		**38**
	**Soil 35 d**		**Soil 35 d**		**Soil 35 d**	
Fresh weight [mg]	528	427	605	456	619	448
SD	127	86	104	74	87	109
**% WT**		**81**		**75**		**72**

Plants were grown in soil in six independent experiments. Student's t-test *P*-values were below 0.05 in all experiments.

Supplement to figure 5. Relative growth of wild-type, *exo*, and complemented *exo* plants (carrying the 35S::EXOga construct) in response to BL-treatments.

	Cotyledon length			Cotyledon width		
	**WT**	***exo***	**EXOga in *exo***	**WT**	***exo***	**EXOga in *exo***
**0 nM BL**						
Mean	100	100	100	100	100	100
SD	11	11	11	17	11	10
**1 nM BL**						
Mean	114	107	119	102	102	110
SD	14	8	15	11	10	11
**10 nM BL**						
Mean	122	109	128	110	99	110
SD	16	10	14	17	9	12
**100 nM BL**						
Mean	119	105	124	100	98	110
SD	14	9	17	14	7	16

	Hypocotyl length		
	**WT**	***exo***	***EXO*ga in *exo***
**0 nM BL**			
Mean	100	100	100
SD	14	9	9
**1 nM BL**			
Mean	121	100	117
SD	16	17	24
**10 nM BL**			
Mean	163	115	135
SD	23	16	24
**100 nM BL**			
Mean	197	170	176
SD	19	28	21

	Root length		
	WT	*exo*	***EXO*ga in *exo***
**0 nM BL**			
Mean	100	100	100
SD	7	14	8
**1 nM BL**			
Mean	48	20	43
SD	11	5	12
**10 nM BL**			
Mean	42	18	34
SD	10	5	9
**100 nM BL**			
Mean	37	12	36
SD	11	4	9

BL treatments were compared to the respective control treatment (0 nM BL = 100%).

Supplement to figure 5. Statistical analysis (ANOVA).

	Cotyledon length		Cotyledon width		Hypocotyl length		Root length	
	**F-value**	**P-value**	**F-value**	**P-value**	**F-value**	**P-value**	**F-value**	**P-value**
Genotype	544,634	<0,001	306,826	<0,001	15,064	<0,001	791,265	<0,001
Treatment	71,127	<0,001	8,366	<0,001	540,574	<0,001	2163,428	<0,001
Genotype x treatment	9,049	<0,001	4,541	<0,001	11,261	<0,001	5,827	<0,001

The ANOVA test was performed using the Holm-Sidak test.
